# Crystal structure of 2,4,6-tri­methyl­benzoic anhydride

**DOI:** 10.1107/S2056989017014670

**Published:** 2017-10-20

**Authors:** Michael A. Land, Katherine N. Robertson, Jason A. C. Clyburne

**Affiliations:** a Atlantic Centre for Green Chemistry, Department of Chemistry, Saint Mary’s University, 923 Robie Street, Halifax, Nova Scotia, B3H 3C3 Canada

**Keywords:** crystal structure, 2,4,6-tri­methyl­benzoic anhydride, mesitoic anhydride

## Abstract

The title compound, C_20_H_22_O_3_, was formed in the reaction between 2,4,6-tri­methyl­benzoic acid and *N*,*N*-diiso­propyl­ethyl­amine in the presence of 1,3-di­chloro-1,3-bis­(di­methyl­amino)­propenium hydrogen dichloride, and was recrystallized from diethyl ether solution. It is the first exclusively alkyl-substituted benzoic anhydride to have been structurally characterized. The asymmetric unit consists of a half mol­ecule, the other half of which is generated by twofold rotation symmetry; the dihedral angle between the symmetry-related aromatic rings is 54.97 (3)°. The geometric parameters of the aromatic ring are typical of those for 2,4,6-tri­methyl­phenyl substituted groups. The C=O and C—O bond lengths are 1.1934 (12) and 1.3958 (11) Å, respectively, and the angle between these three atoms (O=C—O) is 121.24 (9)°. In the crystal, mol­ecules are linked by weak C—H⋯O hydrogen bonds and C—H⋯π inter­actions. The packing features wavy chains that extend parallel to [001].

## Abstract 2

The title compound was isolated as a by-product from a reaction incorporating 2,4,6-tri­methyl­benzoic acid under basic conditions.

## Chemical context   

Benzoic anhydrides have traditionally been used in synthetic organic chemistry for the preparation of aromatic esters, amides and carb­oxy­lic acids. Aromatic anhydrides have also been shown to be effective acyl­ating agents (Shiina, 2004 ▸; Shiina & Nakata, 2007 ▸). The title compound has been used to trap deprotonated 3,4-ep­oxy-2,3,4,5-tetra­hydro­thio­phene 1,1-dioxide, forming 3-(2,4,6-tri­methyl­benzo­yloxy)-2,3-di­hydro­thio­phene 1,1-dioxide (Alonso *et al.*, 2004 ▸, 2005 ▸). The synthesis of the compound we report here, 2,4,6-tri­methyl­benzoic anhydride (common name: mesitoic anhydride), was first published in 1941, where it was formed in the reaction between (2,4,6-tri­methyl­phenyl)sodium and 2,4,6-tri­methyl­benzoic acid in the presence of pyridine (Fuson *et al.*, 1941 ▸). Recently, several new approaches for the syntheses of symmetric acid anhydrides, including the title compound, have been reported (Kazemi *et al.*, 2004 ▸; Li *et al.*, 2012 ▸; McCallum & Barriault, 2015 ▸). The most recent report involves the *in situ* generation of a Vilsmeier–Haack reagent for the coupling of symmetric carb­oxy­lic acids (McCallum & Barriault, 2015 ▸). Due to the structural similarities between this reagent and the 1,3-di­chloro-1,3-bis­(di­methyl­amino)­propenium salt used here, it is possible that the title compound was formed *via* a similar method in our reaction. The crystal structure that we report is the first example of a benzoic anhydride where the aryl rings are substituted with only alkyl groups, although several other substituted benzoic anhydrides are known.
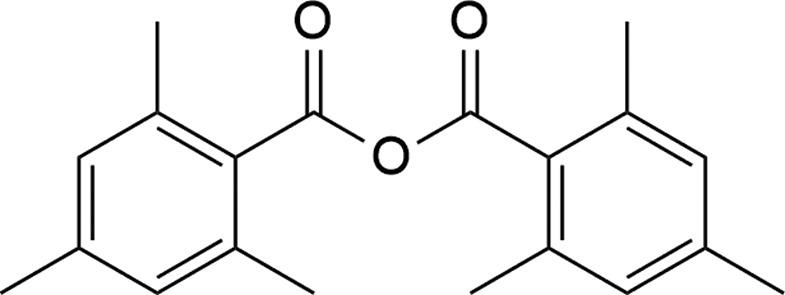


## Structural commentary   

**Figure 1:**
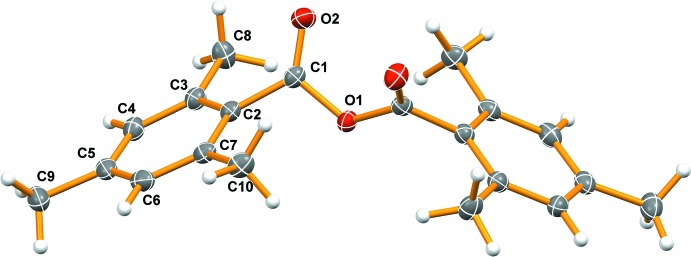
The mol­ecular structure of the title compound. Only half of the mol­ecule is crystallographically unique (labelled atoms). Displacement ellipsoids are drawn at the 50% probability level.

The mol­ecular structure of the title compound is shown in Fig. 1 ▸. It crystallizes in the monoclinic space group *C*2/*c* with one half of the mol­ecule uniquely present in the asymmetric unit. The two C—O bond lengths are significantly different, as would be expected for anhydrides, with lengths of 1.1934 (12) (C1—O2) and 1.3958 (11) Å (C1—O1). The C1—C2 distance is normal for an *sp* ^2^–*sp* ^2^ bond, with a length of 1.4873 (13) Å. The second half of the mol­ecule, which is generated by rotation about the twofold axis passing through O1 (0, *y*, 0.25), forms a dihedral angle of 54.97 (3)° between the equivalent aromatic rings. If the planes of the two overlapping CO_2_ groups are chosen instead, the dihedral angle becomes 59.30 (11)°. The C—C bonds in the aromatic ring are not all statistically equivalent. Unsurprisingly, the longest C—C bonds in the ring are adjacent to the electron-withdrawing anhydride group, C2—C3 [1.4032 (13) Å] and C2—C7 [1.4059 (13) Å]. The remaining C—C bonds are statistically equivalent, averaging 1.3942 (8) Å. All of the C—CH_3_ bond lengths are statistically equivalent with an average length of 1.5102 (8) Å.

## Supra­molecular features   

**Figure 2:**
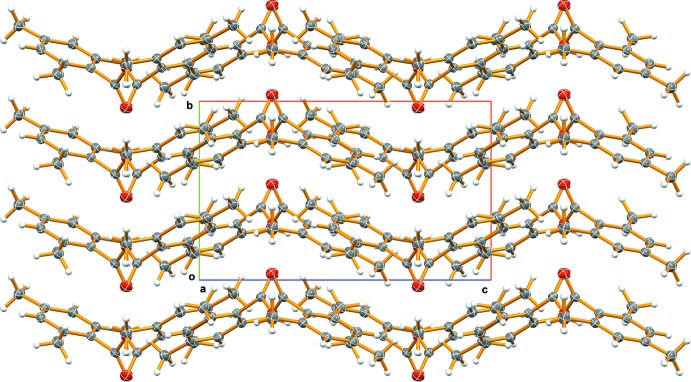
Packing diagram of the title compound, viewed in projection down [100], showing wavy [001] chains.

The packing of the mol­ecules, when viewed in projection down the *a* axis, forms wavy chains that run parallel to the *c*-axis direction (Fig. 2 ▸). Within the chains, the mol­ecules are oriented in a alternating up and down fashion, shifting by 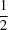 along [001] each time, such that they overlap slightly. There are no close stacking inter­actions between the phenyl rings in various planes. However, if the packing is viewed down the *b* axis, the C5—C6—C7—C10 fragment of one tri­methyl­phenyl group lies directly above/below the same fragment running in the opposite direction, C10—C7—C6—C5, in the plane above/below it.

There are short intra­molecular contacts between the aromatic H atoms H4 (2.37 Å to H8*A* and 2.37 to H9*C*) and H6 (2.39 Å to H10*A*), and the designated methyl H atoms, which close five-membered rings in the mol­ecule.

**Figure 3:**
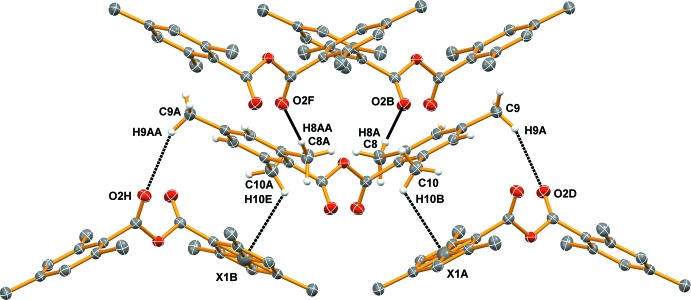
Short inter­molecular contacts (defined in the text and close to the sum of the van der Waals radii; shown as heavy dotted lines), with only donors from the central mol­ecule included (Table 1 ▸).

In the crystal, mol­ecules are linked by weak inter­molecular C—H⋯O hydrogen bonds and C—H⋯π contacts (Table 1 ▸, Fig. 3 ▸). It is notable that these contacts involve one H atom from each of the three methyl substituents on the phenyl ring. All of these contacts occur between the chains that run parallel to the *c* axis and not within the individual chains, thus consolidating the overall structure.

## Database survey   

A survey of the Cambridge Structural Database (CSD, Version 5.38; Groom *et al.*, 2016 ▸), performed on 24 July, 2017, located 25 substituted benzoic anhydrides, all of which were symmetric. Inter­estingly, there were no other benzoic an­hydrides identified that were substituted exclusively with alkyl groups. The only other structurally characterized alkyl-substituted benzoic anhydrides are 2-acet­oxy-5-methyl­benzoic anhydride (CSD refcode IBOCOT; Solanko & Bond, 2011 ▸) and 2-methyl-3-nitro­benzoic anhydride (QUFTIW; Moreno-Fuquen *et al.*, 2015 ▸). Most of the examples found in the CSD contain various substitution patterns involving halogens (Cl, Br or I). There are also several structures that contain aromatic activating groups, such as ethers or amines. The parent compound, benzoic anhydride (ZZZQRI; van Alen & Krauze, 1964 ▸), is known, as is the precursor to the title compound, 2,4,6-tri­methyl­benzoic acid (TMBZAC; Florencio & Smith, 1970 ▸), which can be overlaid with the asymmetric unit of the title compound reasonably well.

## Synthesis and crystallization   

The title compound was isolated from the following reaction mixture, although more convenient synthetic methods are known (Fuson *et al.*, 1941 ▸; Kazemi *et al.*, 2004 ▸). 2,4,6-Tri­methyl­benzoic acid (1.11 g, 6.78 mmol) and *N*,*N*-diiso­propyl­ethyl­amine (1.21 ml, 6.94 mmol) were added to a chloroform solution (25 ml) of 1,3-di­chloro-1,3-bis­(di­methyl­amino)­propenium hydrogen dichloride (0.92 g, 3.42 mmol), which had been prepared following the known literature method (Janousek & Viehe, 1971 ▸), in chloro­form (25 ml). The mixture was stirred at reflux for 18 h under nitro­gen. After cooling to room temperature, a saturated KOH (aqueous) solution (∼2 ml) and water (70 ml) were added to the mixture. The organic layer was extracted and the aqueous phase was washed with chloro­form (two 25 ml portions). The combined organic extracts were washed with brine and dried with MgSO_4_, and the solvent was removed *in vacuo*. The resulting material was washed with ice-cold water (25 ml) and then isolated *via* vacuum filtration. The off-white solid was further purified by recrystallization through slow evaporation of a saturated diethyl ether solution. After 3 h, clear and colourless thin plate-like crystals were obtained [yield: 0.98 g, 3.15 mmol, 93%; m.p. 374–375 K (literature = 375–377 K)]. Elemental analysis, calculated for C_20_H_22_O_3_ (%): C 77.39, H 7.14, N 0.00; found (%): C 77.26, H 7.13, N 0.01. The ^1^H and ^13^C{^1^H} NMR and IR spectroscopic data for the title compound are identical to those previously reported (Kazemi *et al.*, 2004 ▸).

## Refinement   

Crystal data, data collection, and structure refinement details are summarized in Table 2 ▸. H atoms were included in calculated positions (C—H = 0.95–0.98 Å) and refined as riding with *U*_iso_(H) = 1.2 or 1.5*U*_eq_(C).

## Supplementary Material

Crystal structure: contains datablock(s) I, global. DOI: 10.1107/S2056989017014670/hb7710sup1.cif

Structure factors: contains datablock(s) I. DOI: 10.1107/S2056989017014670/hb7710Isup2.hkl

Supporting information file. DOI: 10.1107/S2056989017014670/hb7710Isup3.cml

CCDC reference: 1579203

Additional supporting information:  crystallographic information; 3D view; checkCIF report

## Figures and Tables

**Table 1:** Hydrogen-bond geometry (Å, °) *Cg*1 is the centroid of the C2–C7 ring.

*D*—H⋯*A*	*D*—H	H⋯*A*	*D*⋯*A*	*D*—H⋯*A*
C8—H8*A*⋯O2^i^	0.98	2.48	3.4612 (13)	176
C9—H9*A*⋯O2^ii^	0.98	2.66	3.5842 (14)	157
C10—H10*B*⋯*Cg*1^iii^	0.98	2.96	3.5255 (14)	118

Symmetry codes: (i) 
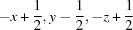
; (ii) 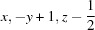; (iii) 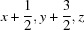.

**Table 2:** Experimental details

Crystal data	
Chemical formula	C_20_H_22_O_3_
*M*_r_	310.37
Crystal system, space group	Monoclinic, *C*2/*c*
Temperature (K)	125
*a*, *b*, *c* (Å)	16.080 (2), 7.9997 (12), 14.308 (2)
β (°)	114.094 (2)
*V* (Å^3^)	1680.1 (4)
*Z*	4
Radiation type	Mo *K*α
μ (mm^−1^)	0.08
Crystal size (mm)	0.55 × 0.26 × 0.25

Data collection	
Diffractometer	Bruker APEXII CCD area-detector
Absorption correction	Multi-scan (*SADABS*; Bruker, 2008 ▸)
*T*_min_, *T*_max_	0.683, 0.746
No. of measured, independent and observed [*I* > 2σ(*I*)] reflections	9832, 2095, 1857
*R*_int_	0.019
(sin θ/λ)_max_ (Å^−1^)	0.679

Refinement	
*R*[*F* ^2^ > 2σ(*F* ^2^)], *wR*(*F* ^2^), *S*	0.037, 0.107, 1.05
No. of reflections	2095
No. of parameters	108
H-atom treatment	H-atom parameters constrained
Δρ_max_, Δρ_min_ (e Å^−3^)	0.34, −0.19

Computer programs: *APEX2* and *SAINT* (Bruker, 2008 ▸), *SHELXT2014* (Sheldrick, 2015*a* ▸), *SHELXL2014* (Sheldrick, 2015*b* ▸) and *Mercury CSD 3.9* (Macrae *et al.*, 2008 ▸).

## supplementary crystallographic information

### Crystal data

**Table:**

C_20_H_22_O_3_	*F*(000) = 664
*M**_r_* = 310.37	*D*_x_ = 1.227 Mg m^−^^3^
Monoclinic, *C*2/*c*	Mo *K*α radiation, λ = 0.71073 Å
*a* = 16.080 (2) Å	Cell parameters from 6207 reflections
*b* = 7.9997 (12) Å	θ = 2.8–28.7°
*c* = 14.308 (2) Å	µ = 0.08 mm^−^^1^
β = 114.094 (2)°	*T* = 125 K
*V* = 1680.1 (4) Å^3^	Wedge shaped (cut from a large block), colourless
*Z* = 4	0.55 × 0.26 × 0.25 mm

### Data collection

**Table:**

Bruker APEXII CCD area-detector diffractometer	2095 independent reflections
Radiation source: sealed tube	1857 reflections with *I* > 2σ(*I*)
Graphite monochromator	*R*_int_ = 0.019
φ and ω scans	θ_max_ = 28.9°, θ_min_ = 2.8°
Absorption correction: multi-scan (SADABS; Bruker, 2008)	*h* = −21→21
*T*_min_ = 0.683, *T*_max_ = 0.746	*k* = −10→10
9832 measured reflections	*l* = −19→19

### Refinement

**Table:**

Refinement on *F*^2^	Primary atom site location: structure-invariant direct methods
Least-squares matrix: full	Hydrogen site location: inferred from neighbouring sites
*R*[*F*^2^ > 2σ(*F*^2^)] = 0.037	H-atom parameters constrained
*wR*(*F*^2^) = 0.107	*w* = 1/[σ^2^(*F*_o_^2^) + (0.0543*P*)^2^ + 0.9964*P*] where *P* = (*F*_o_^2^ + 2*F*_c_^2^)/3
*S* = 1.05	(Δ/σ)_max_ < 0.001
2095 reflections	Δρ_max_ = 0.34 e Å^−^^3^
108 parameters	Δρ_min_ = −0.19 e Å^−^^3^
0 restraints	

### Special details

**Table:**

Geometry. All esds (except the esd in the dihedral angle between two l.s. planes) are estimated using the full covariance matrix. The cell esds are taken into account individually in the estimation of esds in distances, angles and torsion angles; correlations between esds in cell parameters are only used when they are defined by crystal symmetry. An approximate (isotropic) treatment of cell esds is used for estimating esds involving l.s. planes.

### Fractional atomic coordinates and isotropic or equivalent isotropic displacement parameters (Å^2^)

**Table:**

	*x*	*y*	*z*	*U*_iso_*/*U*_eq_	
O1	0.0000	0.31743 (12)	0.2500	0.0206 (2)	
O2	0.08932 (5)	0.53524 (10)	0.24991 (6)	0.0272 (2)	
C1	0.05461 (6)	0.40599 (12)	0.21238 (7)	0.0193 (2)	
C2	0.06727 (6)	0.31349 (12)	0.12897 (7)	0.0190 (2)	
C3	0.15548 (7)	0.26116 (12)	0.14551 (7)	0.0206 (2)	
C4	0.16788 (7)	0.18095 (13)	0.06542 (8)	0.0229 (2)	
H4	0.2269	0.1425	0.0759	0.028*	
C5	0.09582 (8)	0.15592 (13)	−0.02937 (8)	0.0237 (2)	
C6	0.00895 (7)	0.20904 (13)	−0.04303 (8)	0.0245 (2)	
H6	−0.0406	0.1912	−0.1073	0.029*	
C7	−0.00725 (7)	0.28740 (12)	0.03480 (8)	0.0213 (2)	
C8	0.23514 (7)	0.28879 (15)	0.24696 (8)	0.0275 (2)	
H8A	0.2859	0.2167	0.2514	0.041*	
H8B	0.2170	0.2615	0.3028	0.041*	
H8C	0.2542	0.4061	0.2526	0.041*	
C9	0.11059 (9)	0.07659 (15)	−0.11716 (9)	0.0310 (3)	
H9A	0.1003	0.1600	−0.1709	0.047*	
H9B	0.0679	−0.0164	−0.1449	0.047*	
H9C	0.1732	0.0347	−0.0926	0.047*	
C10	−0.10199 (7)	0.34744 (15)	0.01547 (8)	0.0268 (2)	
H10A	−0.1368	0.3649	−0.0581	0.040*	
H10B	−0.0980	0.4530	0.0519	0.040*	
H10C	−0.1326	0.2634	0.0401	0.040*	

### Atomic displacement parameters (Å^2^)

**Table:**

	*U*^11^	*U*^22^	*U*^33^	*U*^12^	*U*^13^	*U*^23^
O1	0.0218 (5)	0.0187 (5)	0.0253 (5)	0.000	0.0138 (4)	0.000
O2	0.0251 (4)	0.0256 (4)	0.0340 (4)	−0.0059 (3)	0.0153 (3)	−0.0060 (3)
C1	0.0149 (4)	0.0210 (5)	0.0220 (5)	0.0018 (3)	0.0074 (4)	0.0031 (4)
C2	0.0187 (5)	0.0190 (4)	0.0200 (5)	−0.0001 (3)	0.0086 (4)	0.0023 (3)
C3	0.0200 (5)	0.0211 (5)	0.0212 (5)	0.0013 (4)	0.0089 (4)	0.0026 (4)
C4	0.0242 (5)	0.0219 (5)	0.0257 (5)	0.0023 (4)	0.0133 (4)	0.0025 (4)
C5	0.0328 (6)	0.0182 (5)	0.0232 (5)	−0.0022 (4)	0.0146 (4)	0.0011 (4)
C6	0.0271 (5)	0.0238 (5)	0.0199 (5)	−0.0043 (4)	0.0067 (4)	0.0013 (4)
C7	0.0200 (5)	0.0208 (5)	0.0222 (5)	−0.0014 (4)	0.0076 (4)	0.0037 (4)
C8	0.0194 (5)	0.0369 (6)	0.0243 (5)	0.0060 (4)	0.0068 (4)	−0.0018 (4)
C9	0.0439 (7)	0.0273 (5)	0.0270 (5)	−0.0031 (5)	0.0197 (5)	−0.0032 (4)
C10	0.0188 (5)	0.0311 (6)	0.0267 (5)	−0.0002 (4)	0.0053 (4)	0.0039 (4)

### Geometric parameters (Å, º)

**Table:**

O1—C1^i^	1.3958 (11)	C6—C7	1.3921 (15)
O1—C1	1.3958 (11)	C6—H6	0.9500
O2—C1	1.1934 (12)	C7—C10	1.5111 (14)
C1—C2	1.4873 (13)	C8—H8A	0.9800
C2—C3	1.4032 (13)	C8—H8B	0.9800
C2—C7	1.4059 (13)	C8—H8C	0.9800
C3—C4	1.3968 (14)	C9—H9A	0.9800
C3—C8	1.5095 (14)	C9—H9B	0.9800
C4—C5	1.3924 (15)	C9—H9C	0.9800
C4—H4	0.9500	C10—H10A	0.9800
C5—C6	1.3953 (16)	C10—H10B	0.9800
C5—C9	1.5101 (14)	C10—H10C	0.9800
			
C1^i^—O1—C1	119.00 (11)	C6—C7—C10	120.03 (9)
O2—C1—O1	121.24 (9)	C2—C7—C10	121.97 (9)
O2—C1—C2	126.87 (9)	C3—C8—H8A	109.5
O1—C1—C2	111.76 (8)	C3—C8—H8B	109.5
C3—C2—C7	121.65 (9)	H8A—C8—H8B	109.5
C3—C2—C1	118.24 (8)	C3—C8—H8C	109.5
C7—C2—C1	120.03 (9)	H8A—C8—H8C	109.5
C4—C3—C2	118.14 (9)	H8B—C8—H8C	109.5
C4—C3—C8	120.45 (9)	C5—C9—H9A	109.5
C2—C3—C8	121.40 (9)	C5—C9—H9B	109.5
C5—C4—C3	121.63 (9)	H9A—C9—H9B	109.5
C5—C4—H4	119.2	C5—C9—H9C	109.5
C3—C4—H4	119.2	H9A—C9—H9C	109.5
C4—C5—C6	118.69 (9)	H9B—C9—H9C	109.5
C4—C5—C9	121.31 (10)	C7—C10—H10A	109.5
C6—C5—C9	119.99 (10)	C7—C10—H10B	109.5
C7—C6—C5	121.91 (9)	H10A—C10—H10B	109.5
C7—C6—H6	119.0	C7—C10—H10C	109.5
C5—C6—H6	119.0	H10A—C10—H10C	109.5
C6—C7—C2	117.96 (9)	H10B—C10—H10C	109.5
			
C1^i^—O1—C1—O2	35.04 (7)	C8—C3—C4—C5	−179.10 (10)
C1^i^—O1—C1—C2	−148.96 (8)	C3—C4—C5—C6	−1.62 (15)
O2—C1—C2—C3	59.63 (14)	C3—C4—C5—C9	177.01 (9)
O1—C1—C2—C3	−116.09 (9)	C4—C5—C6—C7	0.66 (15)
O2—C1—C2—C7	−117.38 (11)	C9—C5—C6—C7	−177.99 (9)
O1—C1—C2—C7	66.89 (11)	C5—C6—C7—C2	0.47 (15)
C7—C2—C3—C4	−0.20 (14)	C5—C6—C7—C10	178.17 (9)
C1—C2—C3—C4	−177.17 (9)	C3—C2—C7—C6	−0.70 (14)
C7—C2—C3—C8	−179.70 (9)	C1—C2—C7—C6	176.21 (9)
C1—C2—C3—C8	3.33 (14)	C3—C2—C7—C10	−178.35 (9)
C2—C3—C4—C5	1.39 (15)	C1—C2—C7—C10	−1.44 (14)

Symmetry code: (i) −*x*, *y*, −*z*+1/2.

### Hydrogen-bond geometry (Å, º)

Cg1 is the centroid of the C2–C7 ring.

**Table:**

*D*—H···*A*	*D*—H	H···*A*	*D*···*A*	*D*—H···*A*
C8—H8*A*···O2^ii^	0.98	2.48	3.4612 (13)	176
C9—H9*A*···O2^iii^	0.98	2.66	3.5842 (14)	157
C10—H10*B*···*Cg*1^iv^	0.98	2.96	3.5255 (14)	118

Symmetry codes: (ii) −*x*+1/2, *y*−1/2, −*z*+1/2; (iii) *x*, −*y*+1, *z*−1/2; (iv) *x*+1/2, *y*+3/2, *z*.
